# Editorial: Contribution of the maternal microbiome to offspring health

**DOI:** 10.3389/fnut.2023.1229447

**Published:** 2023-06-19

**Authors:** Alfonso Benítez-Páez

**Affiliations:** Host-Microbe Interactions in Metabolic Health Laboratory, Principe Felipe Research Center (CIPF), Valencia, Spain

**Keywords:** human gut microbiota, maternal microbiome, infant health, nutrition, offspring

Prenatal, newborn, and infant health statuses are being actively assessed as they are pointed out as important predictive factors of illness in adulthood, especially for high-prevalent non-communicable diseases and immune disorders. A plethora of factors affecting such conditions are under the spotlight, and the maternal microbiome is one of the most intriguing aspects conditioning the offspring's health. For years, the sterile womb hypothesis has been challenged to explain how the maternal microbiota could reach the placenta and directly affect the infant's health via in-utero colonization. However, some independent studies have revealed methodological issues on the placenta microbiota hypothesis, and others have demonstrated fetal gut colonization of healthy term infants does not occur before birth (1–5). With the sterile womb hypothesis apparently solved, hopefully, the maternal microbiome is still a matter of investigation as a determinant of the offspring's health before and after childbirth. On the one hand, multiple environmental factors shape the maternal microbiome during pregnancy, making possible fetus exposure to a wide variety of metabolic (by)products derived from microbes (essentially in the gut) and circulating systemically in the mother and affecting it in different ways (6). On the other hand, given the strong dependency of newborn feeding on the mother, the human milk microbiome and the perinatal factors conditioning it are also subjects of the investigation to determine early or late onset disease risk factors (7), taking into account also its nutritional relevance for infant's health (8).

The aim of this Frontiers in Nutrition Research Topic (RT) “*Contribution of the maternal microbiome to offspring health*” was to assemble clinical and pre-clinical studies describing insights into the maternal microbiome and its potential effects on offspring health. It is provided an overview of this RT, including two original research articles, one review article, and one study protocol.

## Prebiotics and probiotics to breaking transgenerational obesity

Maternal obesity is understood as a relevant factor in increasing the child's obesity risk, and Wiedmer and Herter-Aeberli show a narrative review of nutritional strategies to reduce obese pregnancy as a promising strategy to tackle this transgenerational diet-related disorder and comorbidities. The authors highlight the meaningful impact of short-chain fatty acids SCAFs, produced by gut microbes, on maternal and offspring health. Notably, in this last, the embryonic differentiation of enteroendocrine cells would be influenced by the action of SCFAs on the GPR43 receptor, making high-fiber dietary regimes a protector factor against predisposed adiposity. After a detailed assessment of the pre-clinical and clinical evidence published, the efficacy of probiotics in managing weight gain and metabolic function in mothers and offspring is blurred in both types of studies, probably due to the variability of trials and utilization of different probiotic formulations. However, prebiotic administration showed promising results, especially in the offspring's body weight control and metabolic function.

## Human milk microbiome composition upon SARS-CoV-2 infection

It's important to be aware of the cornerstone role of human milk as a nutrient source, immune defense, and as the seed of the infants' gut microbiota, thus influencing their health by extension. In this context, Gómez-Torres et al. have measured the impact of SARS-CoV-2 infection in the bacterial composition of human milk to unveil potential mid- and long-term alterations in offspring health as a consequence of the COVID-19 pandemic. This multicentre clinical study explored the human milk microbiota profiles of more than 140 mothers who decided to breastfeed their healthy babies regardless of whether they were positive or negative for SARS-CoV-2 infection at delivery. One week and 5 weeks postpartum milk samples were negative for detection of SARS-CoV-2, and they were assessed using standard 16S rRNA gene sequencing approaches. After recording critical perinatal variables for integration, the authors describe minimal impact of SARS-CoV-2 infection on human milk microbiota composition, getting similar patterns observed in other populations upon evaluating non-infected individuals.

## Human milk-borne pathogen alters offspring immunity

This RT compiles another intriguing study trying to decipher the effects of pathogens transmitted from mothers to offspring via breastfeeding, thus with the potential to alter the health status and development of progeny. Morales-Ferré et al. present a comprehensive study on the immune profiling of rat pups upon oral administration of low-load and high-load *Staphylococcus epidermis* (a human mastitis pathogen) during lactation period. Despite the fact that the rat model of exposure to mastitis pathogen showed no changes in terms of offspring weight gain or intestinal physiology, but sustained oral administration of *S. epidermis* for 21 days (lactation period) does alter the circulating lymphocyte population as well as reduce the plasma Ig levels. Besides, exposure to *S. epidermis* during the lactation period also seems to drive an unbalance of Th1:Th2 signaling with potential short- and long-term effects on rat pups to clear infections and regulate immune response.

## Disentangling the microbiota mediated mother-to-children interactions

The infants' early life is critical to the development and health programming, resulting in long-lasting effects conditioning predisposition to a wide variety of infectious and non-communicable diseases as well as behavioral and mood dysfunctions. Consequently, Eow et al. propose an ambitious follow-up study (MYBIOTA) of mother-infant pairs during 12 months postpartum, in Malaysia, where they will collect and assess a wide array of clinical host-associated and molecular-derived data to understand the host-microbe interactions in the first year of life and disclose mother-derived and environmental determinants with high impact on the offspring health. Furthermore, with the longitudinal perspective, this study will also have the capacity to unveil the effects of such determinants on the development of fetuses, newborns and infants. All in all, this study will promise to fill the knowledge gap on the colonization and development of the gut microbiome during early life and to understand how it depends on mother interaction from the molecular and microbiological point of view.

The contributions gathered in this RT represent a clear advance in delineating how the maternal microbiome can modulate the offspring's health, paving the way for future studies and clinical application of the insights compiled here.

## Author contributions

AB-P read all the contributions to the Research Topic and prepared this editorial.
